# Sheep-Specific Immunohistochemical Panel for the Evaluation of Regenerative and Inflammatory Processes in Tissue-Engineered Heart Valves

**DOI:** 10.3389/fcvm.2018.00105

**Published:** 2018-08-15

**Authors:** Sylvia Dekker, Daphne van Geemen, Antoon J. van den Bogaerdt, Anita Driessen-Mol, Elena Aikawa, Anthal I. P. M. Smits

**Affiliations:** ^1^Soft Tissue Engineering & Mechanobiology Division, Department of Biomedical Engineering, Eindhoven University of Technology, Eindhoven, Netherlands; ^2^Division Heart Valve Bank, ETB-BISLIFE, Beverwijk, Netherlands; ^3^Institute for Complex Molecular Systems (ICMS), Eindhoven University of Technology, Eindhoven, Netherlands; ^4^Division of Cardiovascular Medicine, Department of Medicine, Center for Excellence in Cardiovascular Medicine, Brigham and Women's Hospital, Boston, MA, United States

**Keywords:** immunohistochemistry, ovine antibodies, cardiovascular tissue engineering, elastogenesis, inflammation, macrophages, valvular interstitial cells, extracellular matrix

## Abstract

The creation of living heart valve replacements via tissue engineering is actively being pursued by many research groups. Numerous strategies have been described, aimed either at culturing autologous living valves in a bioreactor (*in vitro*) or inducing endogenous regeneration by the host via resorbable scaffolds (*in situ*). Whereas a lot of effort is being invested in the optimization of heart valve scaffold parameters and culturing conditions, the pathophysiological *in vivo* remodeling processes to which tissue-engineered heart valves are subjected upon implantation have been largely under-investigated. This is partly due to the unavailability of suitable immunohistochemical tools specific to sheep, which serves as the gold standard animal model in translational research on heart valve replacements. Therefore, the goal of this study was to comprise and validate a comprehensive sheep-specific panel of antibodies for the immunohistochemical analysis of tissue-engineered heart valve explants. For the selection of our panel we took inspiration from previous histopathological studies describing the morphology, extracellular matrix composition and cellular composition of native human heart valves throughout development and adult stages. Moreover, we included a range of immunological markers, which are particularly relevant to assess the host inflammatory response evoked by the implanted heart valve. The markers specifically identifying extracellular matrix components and cell phenotypes were tested on formalin-fixed paraffin-embedded sections of native sheep aortic valves. Markers for inflammation and apoptosis were tested on ovine spleen and kidney tissues. Taken together, this panel of antibodies could serve as a tool to study the spatiotemporal expression of proteins in remodeling tissue-engineered heart valves after implantation in a sheep model, thereby contributing to our understanding of the *in vivo* processes which ultimately determine long-term success or failure of tissue-engineered heart valves.

## Introduction

Valvular heart disease is a major health problem. Heart valve tissue engineering (TE) strategies aim to create autologous, living heart valves with the potential for growth and remodeling to replace the malfunctioning heart valve. Since approximately two decades ago, several TE strategies have been actively pursued to create living heart valve replacements *in vitro*, using a variety of cell sources, scaffold types and fabrication methods (1–11). Alternative to the traditional *in vitro* TE paradigm, *in situ* TE strategies are being developed, using acellular, readily-available scaffolds which are designed to induce endogenous tissue regeneration directly at the valve's functional site (12–14). *In situ* TE scaffolds include natural matrices, such as decellularized allografts (15–18) or *de novo* engineered and decellularized heart valves (19–23), or resorbable synthetic heart valves (24, 25).

For all heart valve TE strategies, the main challenge lies in the functional, and eventually homeostatic integration of the TE valve into the body. In particular, mimicking the sophisticated tri-layered structure of the native valve, including a well-organized anisotropic collagen and elastin network populated with quiescent valvular interstitial cells (VICs), requires a thorough understanding of valvular developmental and biomechanical remodeling processes (26, 27). Moreover, the host immune response evoked by a TE heart valve after implantation is perhaps the most crucial determinant of successful valve integration. This is even more important for *in situ* TE strategies, which rely on triggering a favorable inflammatory response upon implantation in order to induce a regenerative cascade (13, 28, 29).

In general, any implanted device (whether or not cellular) will induce an inflammatory response by the host. This response occurs as a cascade of events, ignited by disruption of the tissue structure and cell damage due to the implantation procedure (30). At the very early stages of implantation, blood-biomaterial interactions lead to the adsorption of endogenous proteins from blood to the biomaterial surface. Following this provisional matrix formation and within the first days after implantation, acute inflammation occurs with an immediate influx of innate immune cells, mostly neutrophils and monocytes. In later stages, chronic inflammation develops as inflammatory stimuli persist at the implant side, with phagocytic macrophages and multinucleated giant cells controlling the microenvironment in cross-talk with lymphocytes. Tissue producing cells such as endothelial cells, fibroblasts, and various (circulating) stem and progenitor cells of both mesenchymal and hematopoietic origin, produce an extracellular matrix (ECM) and account for remodeling as in the cascade of normal wound healing. Depending on the implant properties and the host's immune tissue responses, these may lead to either functional regenerated tissue or pathological fibrotic repair (13, 28). In addition, in case of a TE heart valve prosthesis, healing outcome is influenced by the direct exposure to the blood stream and the resulting hemodynamic loads. However, although some of these immunological events have been experimentally demonstrated for *in situ* TE blood vessel prostheses (31, 32), specific information regarding these immunological processes for TE heart valves is sparse.

Heart valve TE research has predominantly focused on optimizing valvular graft design and *in vitro* culture protocols (6–11, 33), and relatively little mechanistic data is available regarding the *in vivo* biological events that drive remodeling and integration of TE valves after implantation (34). *In vivo* testing of heart valves is typically performed in sheep, as this is the FDA-approved animal model for preclinical evaluation of heart valves. The heart valves of sheep resemble those of humans in terms of mechanical properties and hemodynamic flow parameters (35). Furthermore, sheep develop relatively rapidly, thus the growth and remodeling processes to which heart valves are subjected within several months in juvenile sheep, take several years to develop in patients (36). Finally, due to the enhanced calcium metabolism in sheep, the sheep represent the “worst-case-scenario” in terms of valvular calcification (37, 38). For the bulk of *in vivo* studies using TE heart valves, explant analysis is focused on valve functionality, overall tissue composition and mechanical properties. However, there is an imminent need for mechanistic studies to unravel the biological processes underlying *in vivo* valve integration and remodeling in terms of cellular infiltration and phenotypical characterization, immunological processes, tissue formation/organization, and valve development (34).

So far, a detailed spatiotemporal characterization of cellular phenotypes and tissue remodeling is lacking due to the limited commercially available sheep-specific antibodies for immunohistochemistry. Therefore, the goal of this study was to develop a comprehensive sheep-(cross) reactive marker panel of antibodies to assess the inflammatory and regenerative processes in TE heart valves. Based on previous studies on the composition of native human heart valves throughout development and adult stages (39–42), the panel includes antibodies to study ECM composition, elastogenesis, VIC phenotypes, endothelial cells (ECs), proliferation/apoptosis, and inflammation. These antibodies were tested against paraffin sections of native ovine aortic valve tissue. Markers for inflammatory cells and apoptosis were tested on ovine spleen and kidney tissue.

## Methods

### Sheep tissue

The desired control tissues (aortic heart valve, kidney) were collected from two juvenile sheep (female, Swifter, 1 year old). Additionally, the spleen of a juvenile sheep (female, Swifter, 1 year old) diagnosed with endocarditis was collected to study the inflammatory markers. The ovine tissues from these three sheep were discarded tissues from synthetic pulmonary heart valve replacement experiments. This study was carried out in accordance with local and national regulations, approved by the animal ethics committee of the University Medical Center Utrecht. Also, ovine pulmonary valves were obtained from the slaughterhouse. All tissues were immediately fixed in 4% paraformaldehyde for 24 h at 4°C. After fixation the tissues were embedded in paraffin, sectioned with a thickness of 5 μm, applied to a poly-lysine coated slide (Thermo Scientific), and dried overnight in a 37°C incubator.

All antibodies for valve-related proteins (i.e., ECM-related proteins, proteins involved in elastogenesis, and VIC markers) were validated on the ovine aortic valves (*n* = 2) and ovine pulmonary valve leaflets (*n* = 2). The antibodies for inflammatory proteins were validated on the ovine spleen tissue, representing a tissue with a well-known composition of immune cells.

### Human tissue

Cryopreserved human aortic and pulmonary valves were obtained from a Dutch postmortem donor (male; 38 years old), giving permission for research according to national ethical and regulatory guidelines. This study was carried out in accordance with the recommendations of ETB-BISLIFE, division Heart Valve Bank (Beverwijk, the Netherlands), and was conducted conform the principles outlined in the Declaration of Helsinki. The valves were obtained from ETB-BISLIFE, division Heart Valve Bank and were used in a previous study by our research group, aimed at investigating the histopathological and mechanical properties of human heart valves during various stages of life (40). The valves were assessed to be unfit for implantation due to findings that contra-indicated implantation, consisting among others of positive bacteriological sampling, serological findings in the donor and other procedural non-conformities that caused rejection of the donor (e.g., sexual risk behavior and/or risk in drug abuse). The cause of death was not related to valvular disease or condition known to precede valvular disease and both valves were structurally and mechanically unaffected. After thawing, the tissue was fixed overnight in 4% paraformaldehyde, processed, and subsequently embedded in paraffin to prepare sections with a thickness of 10 μm.

### Histology

Tissue slides were deparaffinized in xylene and rehydrated in a graded series of ethanol. A modified Russell-Movat pentachrome stain (43) was performed to visualize overall tissue composition and organization (American MasterTech). ECM components, including collagen (yellow), elastin (black) and glycosaminoglycan/proteoglycans (GAGs; green-blue) were detected as well as cell nuclei (dark-blue/purple) and fibrin (red). Weigert's Iron Hematoxylin (Sigma) and Eosin (Sigma) staining was performed to reveal distinct compartments of spleen tissue.

### Immunohistochemistry

After deparaffinization, antigen retrieval was performed, depending on the used primary antibody (Table 1). For heat-mediated antigen retrieval, the tissue slides were heated in a 96°C water bath for 20 min in modified citrate buffer (pH 6.1, DAKO) or TRIS-EDTA buffer (pH 9.0, DAKO). After heating, slides were slowly cooled down to room temperature (45 min). For enzymatic antigen retrieval, 0.05% pepsin (Sigma) in 10 mM HCl or 0.6 U/ml Proteinase K (Sigma, in 50 mM Tris Base, 1 mM EDTA, 0.5% Triton X-100, pH 8.0) was applied to the tissue slides for 12 min at 37°C. Nonspecific binding was blocked by either goat serum (Invitrogen) or horse serum (Life Technologies) in 1% bovine serum albumin (BSA; Sigma) in phosphate-buffered saline (PBS; Sigma)/0.05% Tween-20 (Merck) for 2 h at room temperature. Primary antibodies were prepared in the optimized concentrations (see Table 1) in 1:10 diluted blocking solution. Tissue slides were incubated overnight at 4°C and thereafter washed with PBS/tween-20. The applied secondary antibodies were either goat-anti-rabbit antibody (DAKO) or horse-anti-mouse antibody (DAKO), both labeled with biotin and diluted 1:500 in PBS/Tween-20. After 2 h of incubation at room temperature the sections were washed with TRIS-buffered saline (TBS)/tween-20. To enhance staining, an ABC-alkaline phosphatase kit (Vector laboratories, VECTASTAIN® ABC-AP Staining Kit) was used for 1 h at room temperature, according to the manufacturer's guidelines. After washing with TBS/tween-20, the slides were exposed to SIGMA FAST™ BCIP/NBT (5-Bromo-4-chloro-3-indolyl phosphate/Nitro blue tetrazolium, pH 9.5; Sigma) for 5–30 min (depending on the intensity of the staining, which was assessed by light microscopy). For counterstaining of cells, nuclear fast red (Sigma) was applied for 3 min. The sections then were dehydrated and mounted in Entellan (Merck). Negative controls were generated with the same procedure omitting the primary antibody during the first incubation.

**Table 1:** Antibodies used for immunohistochemistry.

**Antibody**	**Host**	**Clone**	**Distributor**	**Cat. No**.	**Antigen retrieval**	**Dilution**
**ECM**						
Collagen type 1	Mouse	Col-1, IgG1	Sigma	C2456	Citrate	200
Collagen type 3	Rabbit	Polyclonal	Abcam	Ab7778	Pepsin	250
Periostin	Rabbit	Polyclonal	n.a.	n.a.	Citrate	500
Versican	Rabbit	Polyclonal	Abcam	Ab6994	Proteinase K	200
Biglycan	Rabbit	Polyclonal	Biorbyt	orb100396	Pepsin	150
Decorin	Rabbit	Polyclonal	Biorbyt	orb10520	Tris/EDTA	150
MMP-1	Rabbit	EP1247Y, IgG	Abcam	ab52631	Citrate	200
MMP-2	Mouse	6E3F8, IgG2b	Abcam	ab86607	Proteinase K	400
MMP-9	Rabbit	Polyclonal	Abcam	ab38898	Citrate	200
MMP-13	Rabbit	Polyclonal	Abcam	ab39012	Citrate	200
TGF-β1	Rabbit	Polyclonal	Abcam	ab9758	Citrate	500
TGF-β3	Rabbit	Polyclonal	Novus Biologicals	NB600-1531	Citrate	400
**ELASTOGENESIS**						
(Tropo)elastin	Rabbit	Polyclonal	Abcam	ab21610	Pepsin	400
Fibronectin	Rabbit	Polyclonal	Sigma	F3648	Pepsin	300
Fibrillin-1	Rabbit	Polyclonal	Sigma	HPA017759	Pepsin	100
Fibrillin-2	Rabbit	Polyclonal	Sigma	HPA012853	Pepsin	100
Lysil oxidase (LOX)	Rabbit	Polyclonal	Novus Biologicals	NB100-2530	Tris/EDTA	200
Fibulin-4	Rabbit	Polyclonal	Abonline	abin1701235	Pepsin	200
Fibulin-5	Mouse	3F10A5, IgG1	Abonline	abin1107239	Proteinase K	100
EMILIN-1	Rabbit	Polyclonal	Biorbyt	orb183385	Pepsin	200
**VICs**						
α-SMA	Rabbit	Polyclonal	Abcam	ab5694	Pepsin/citrate	600
Vimentin	Rabbit	D21H3, IgG	Cell Signalling	5741	Citrate	300
SM22	Rabbit	Polyclonal	Abcam	ab14106	Citrate	200
Calponin	Rabbit	Polyclonal	Abcam	ab46794	Pepsin	150
Cadherin-11	Rabbit	Polyclonal	Thermo Scientific	71-7600	Citrate	100
SMemB	Mouse	3H2, IgG2b	Abcam	ab684	Tris/EDTA	200
**VECs**						
CD31 (PECAM-1)	Mouse	CO.3E-1D4, IgG2a	Novus Biologicals	NB100-65900	Pepsin	100
CD34	Rabbit	EP373Y, IgG	Abcam	ab81289	Tris/EDTA	200
VE-Cadherin	Rabbit	Polyclonal	Abcam	ab33168	Citrate	200
von Willebrand factor	Rabbit	Polyclonal	Abcam	ab6994	Citrate	1200
**PROLIFERATION/APOPTOSIS**						
Ki-67	Rabbit	Polyclonal	Thermo Scientific	rb1510-PO	Citrate	200
Cleaved Caspase-3	Rabbit	Polyclonal	Cell Signalling	9661	Citrate	200
**CALCIFICATION**						
Osteocalcin	Rabbit	Polyclonal	Abcam	ab93876	Tris/EDTA	200
**INFLAMMATION**						
CD45	Mouse	1.11.32, IgG1	AbD-serotec	MCA2220GA	None	1000
CD3	Rabbit	Polyclonal	DAKO	A0452	Tris/EDTA	200
CD64	Mouse	3D3, IgG1	Abcam	ab140779	Citrate	200
CSF-1R (CD115)	Rabbit	Polyclonal	Abonline	abin498117	Citrate	200
EMR-1	Rabbit	Polyclonal	Gene Tex	GTX101895	Tris/EDTA	500
iNOS	Rabbit	Polyclonal	Abcam	ab3523	Citrate	400
CCR7 (CD197)	Rabbit	Polyclonal	Abcam	ab32527	Citrate	1200
TNF-a	Rabbit	Polyclonal	Acris	AP20373PU-n	Citrate	300
CD163	Mouse	EdHu-1, IgG1	AbD-serotec	mca1853	Citrate	250
CD200R	Rabbit	Polyclonal	Abonline	Abin737586	Citrate	200
Arginase-1	Rabbit	Polyclonal	Bioss	bs-8585R-Biotin	Citrate	500
IL-10	Rabbit	Polyclonal	Biorbyt	orb221323	Citrate	200
CXCR4 (CD184)	Mouse	44716, IgG2b	RnD systems	MAB172	Citrate	500
CD44	Rabbit	Polyclonal	Abcam	ab24504	Citrate	250

MMP, matrix metalloproteinase; TGF-β, transforming growth factor-β; EMILIN-1, elastin microfibril interfacer 1; α-SMA, α-smooth muscle actin; CSF-1R, colony stimulating factor-1 receptor; EMR-1, EGF-like module-containing mucin-like hormone receptor-like 1; iNOS, inducible nitric oxide synthase; TNF-α, tumor necrosis factor-α; IL-10, interleukin-10: VE-cadherin, vascular endothelial cadherin

### Imaging and representation

Light microscopy images and tile scans were acquired and documented with a Zeiss Axio Observer Z1 microscope using Zeiss ZEN software. The tile scans of one of the ovine aortic valves are displayed as representative samples for all valves tested. To provide a concise semi-quantitative overview of protein expression in the ovine valves, the protein expression was scored (scale 0–4; no expression—high expression, respectively) per region in the valve (arterial wall, hinge region, belly, and free edge of the leaflet) and per valvular layer (fibrosa, spongiosa, and ventricularis).

## Results

### Human and sheep heart valve histopathology

**Figure 1:**
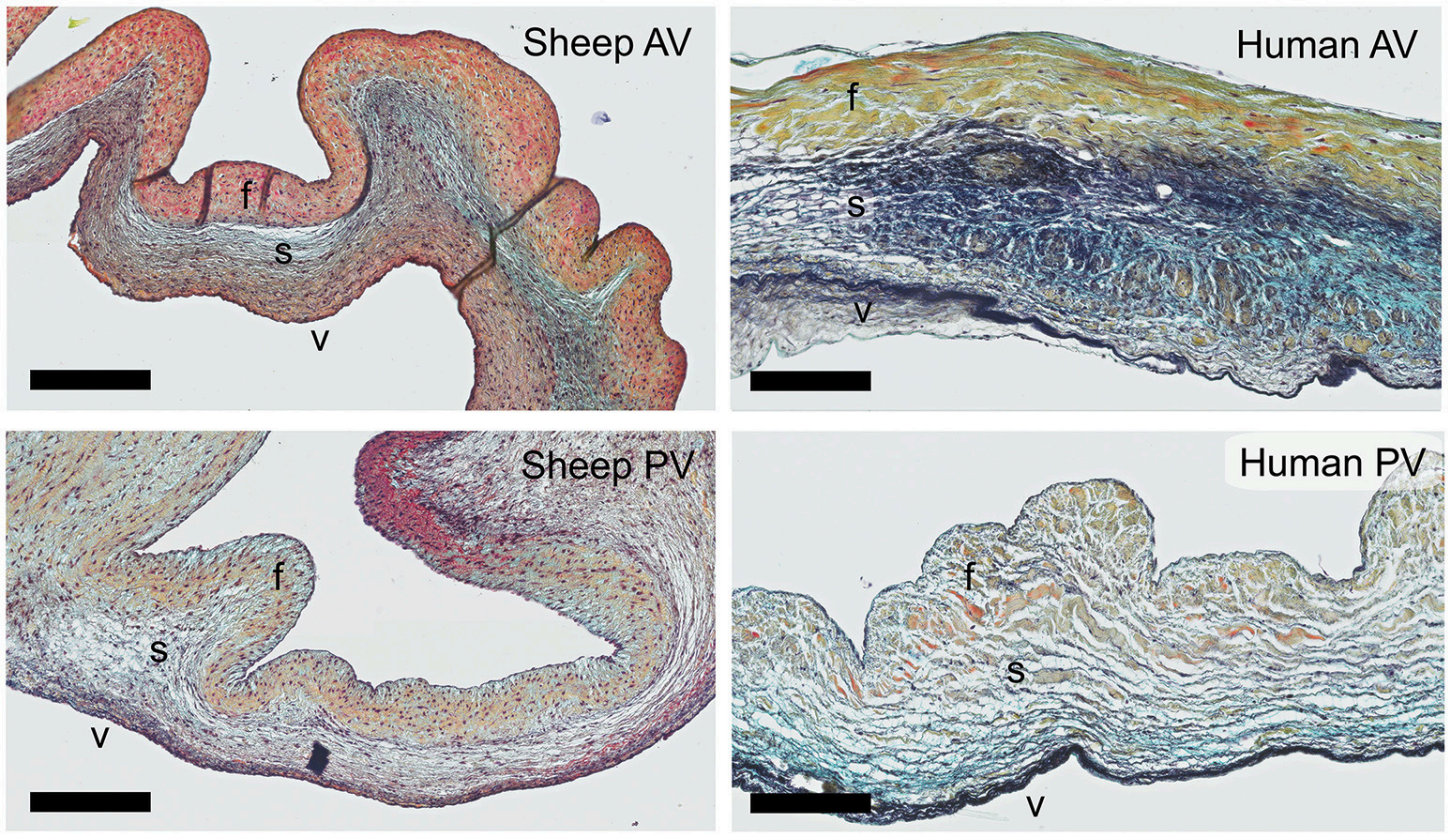
Ovine vs. human heart valves. Russell Movat's pentachrome staining on the sheep aortic valve (AV) and pulmonary valve (PV) (left panel) and their human counterparts (right panel). The images represent sections of the valvular leaflet in the belly region. The valvular layers are indicated in each valve (f, fibrosa; s, spongiosa; v, ventricularis). Scale bars, 200 μm.

Russell-Movat pentachrome staining reveals comparable structure and gross morphology for human and ovine aortic and pulmonary heart valve leaflets, with collagen on the outflow side (fibrosa), elastin on the inflow side (ventricularis) and soluble proteoglycans and glycoproteins in the interstitial layer (spongiosa) (Figure 1). In the sheep valves, the transition from one tissue layer to the other is highly delineated, while in human the layers merge into each other. Toward the free edge of the leaflet, the stratification in the both human and ovine leaflets becomes less pronounced and is no longer observable at the leaflet tip. For both the pulmonary and aortic valves, the ovine leaflets are visibly more cellularized when compared to the human leaflets. Elastin expression shows a remarkable difference in amount and network organization, with a distinct, multi-layered elastic network in the human leaflets, compared to only a few fibers in the sheep leaflets.

### ECM remodeling

**Figure 2:**
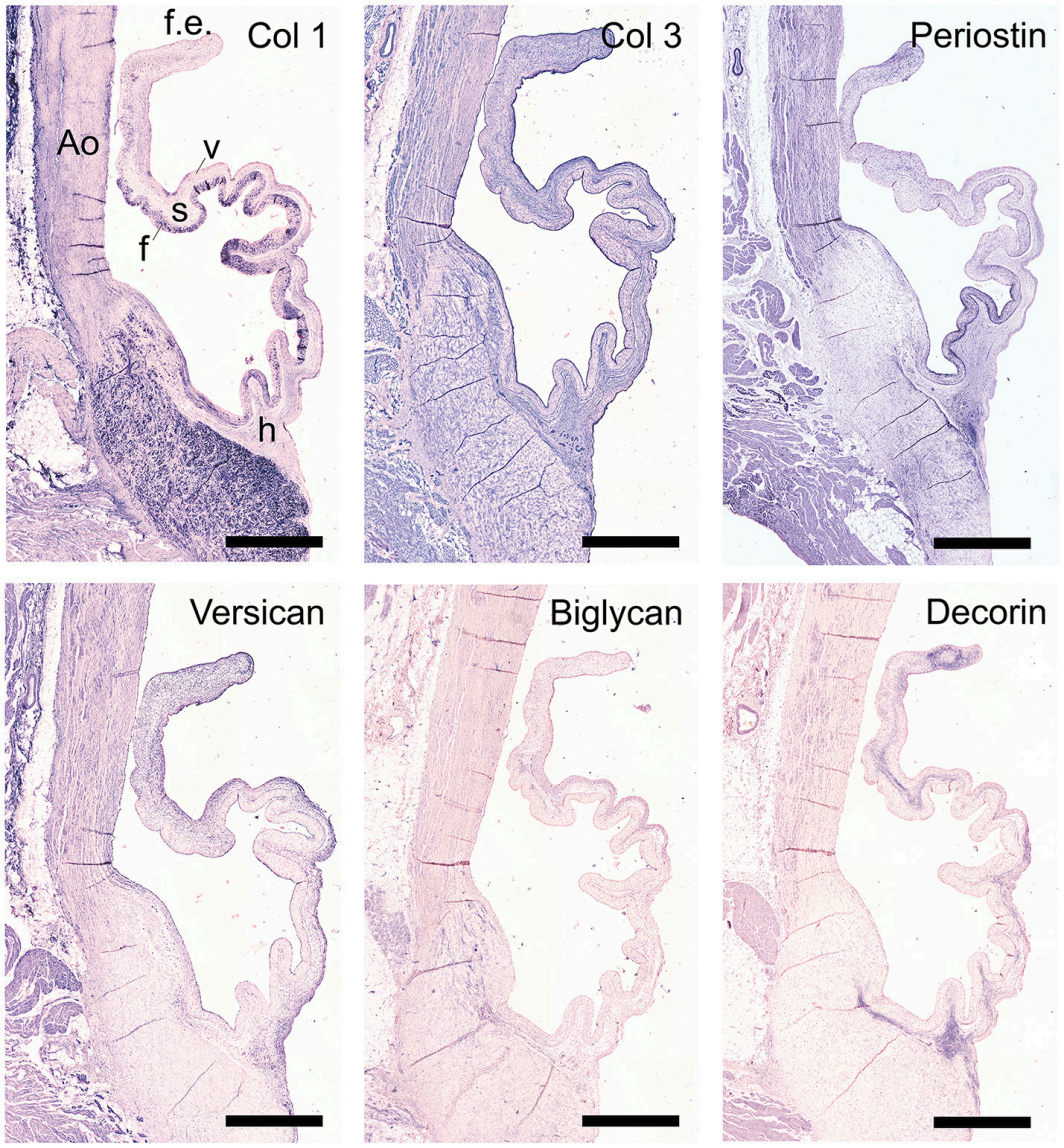
Collagens and proteoglycans. Tile scans of sheep aortic valve sections with the leaflet hinge (h), the free edge (f.e.), the aorta (Ao), and the three valvular layers (f, fibrosa; s, spongiosa; v, ventricularis) as indicated (top left). Valves were stained with antibodies against collagen type 1 (Col 1), collagen type 3 (Col 3), and periostin (top panel), and the proteoglycans versican, biglycan, and decorin (bottom panel). Scale bars, 1 mm.

**Figure 3:**
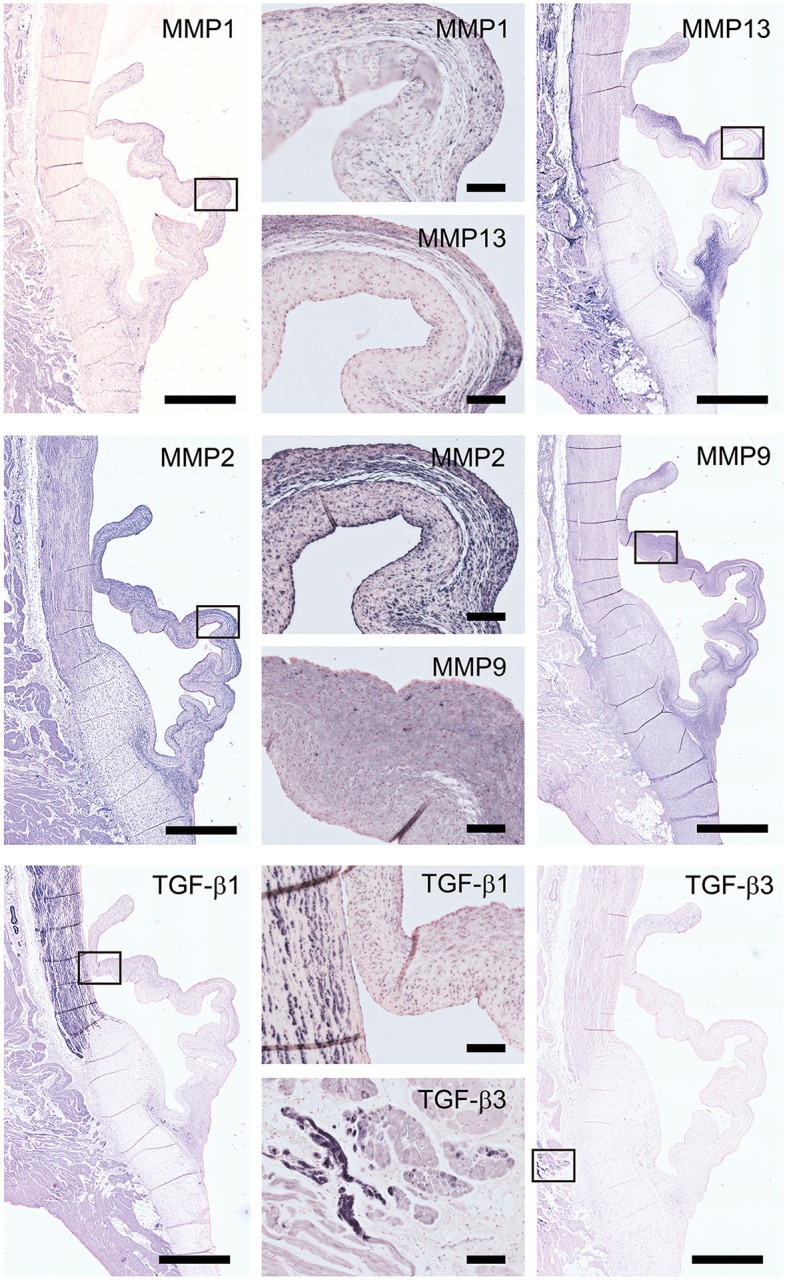
Matrix remodeling and synthesis-related proteins. Tile scans of sheep aortic valve sections (left and right columns), with high magnification zooms (middle column) as indicated. Valves were stained with antibodies against matrix metalloproteinases (MMPs; collagenases MMP1, MMP13, and gelatinases MMP2, MMP9) and transforming growth factor-β, isoforms 1 and 3 (TGF-β1 and TGF-β3, respectively). Scale bars, 1 mm (tile scans) or 100 μm (zooms).

The staining patterns of the major ECM (-related) proteins, including collagen type I and III and protegolycans as well as matrix metalloproteinases (MMPs) and transforming growth factor-β (TGF-β) was analyzed in the ovine aortic valve leaflets (*n* = 2) and pulmonary valve leaflets (*n* = 2). All valves displayed similar staining patterns. Figures 2, 3 display the representative tile scans of protein expression in one of the aortic valves. Negative control sections for all tested antibodies, in which the primary antibody was omitted, revealed no background signal (not shown).

Collagen type I is mainly present in the fibrosal layer in a punctuated pattern indicative of the circumferential orientation of the fibers (Figure 2, top panel). It is also present within the loose connective tissue. Collagen type III expression is overlapping with collagen type I in the fibrosa, but is also present in the spongiosa, endothelial layer, and microvasculature. Periostin is observed in the hinge region, fibrosa, aortic wall, and microvasculature, and is increasingly expressed by interstitial cells toward the free edge of the valve leaflet. Expression of the tested proteoglycans reveals a dispersed distribution of versican throughout the leaflet, as well as connective tissue, microvasculature and cardiac muscle (Figure 2, bottom panel). Biglycan shows an overall weak staining, mainly in the ventricularis. Decorin is predominantly expressed in the spongiosa, with most pronounced expression in the tip and hinge regions.

Matrix metalloproteinases-1 (MMP-1) is predominantly distributed through the spongiosa and is expressed in the microvasculature of the base of the leaflet (Figure 3). MMP-13 is highly present in the spongiosa and ventricularis, the hinge region and the connective tissue. MMP-2 is expressed as an abundant cellular staining throughout the leaflet layers, aorta and microvasculature. MMP-9 is observed in hinge cells and sparsely in cells within the ventricularis. TGFβ1 is strongly expressed in the smooth muscle cell layer of the aortic wall and microvasculature, but not in the valve leaflet, while TGFβ3 shows only a weak staining in the aortic wall (Figure 3).

### Elastic network

**Figure 4:**
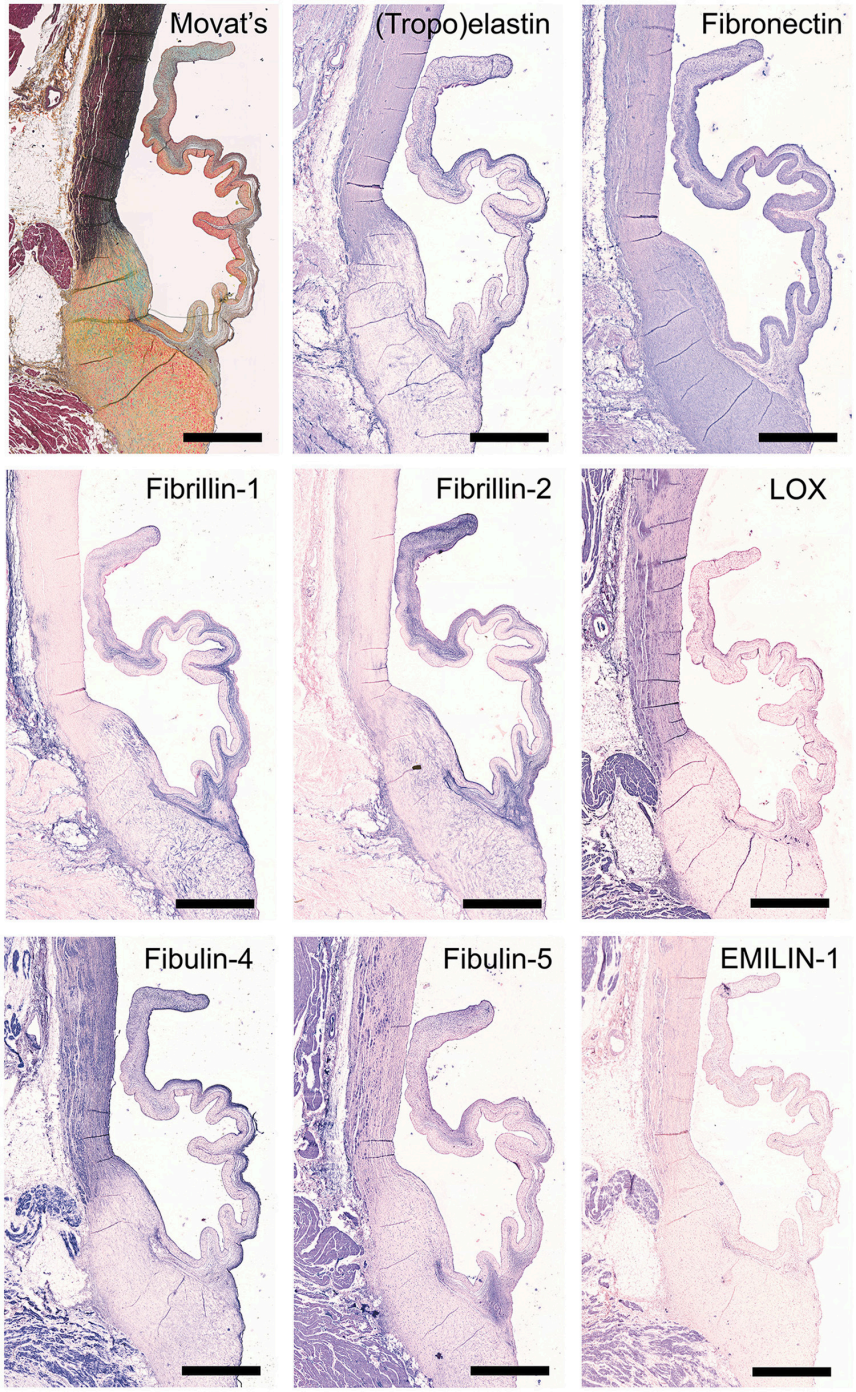
Elastin and elastin-related proteins. Tile scans of sheep aortic valve sections stained with Russell Movat's pentachrome (mature elastic fibers in black) and antibodies against proteins involved in elastic fiber formation, including the core protein (tropo)elastin, fibronectin, the microfibrillar proteins fibrillin-1 and fibrillin-2, and cross-linking proteins fibulin-4, fibulin-5, lysyl oxidase (LOX), and Elastin microfibril interfacer 1 (EMILIN-1). Scale bars, 1 mm.

Movat's shows abundant deposition of elastin in the aortic wall and a weaker staining in the leaflet ventricularis. Selected antibodies to assess elastin-related proteins include (tropo)elastin, fibrillins, fibulins, and cross-linking proteins (Figure 4). (Tropo)elastin is abundant in the ventricularis, but also the endothelial layers and spongiosa are positively stained. Fibronectin is present in all leaflet layers cells, with the most pronounced expression in the fibrosa and ventricularis. Fibrillins (FBN)−1 and−2 are expressed in the spongiosa and the ventricularis. These patterns are mostly overlapping, except for the strong FBN-2 staining in the tip of the leaflet. FBN-2 staining is restricted to the leaflet, while for FBN-1 the heart connective tissue is positive as well. Lysyl oxidase (LOX) exhibits only a mild expression in the ventricularis, while an overall strong staining pattern in the aorta and cardiac muscle tissue is found. Fibulin-4 exhibits strong staining of the ventricularis, as well as the aortic wall, heart tissue, and endothelial cells. For fibulin-5, on the other hand, a mild staining of the spongiosa and ventricularis is observed, in addition to positive staining of VICs in the tip of the leaflet and the endothelial cells of mainly the aorta. Elastin microfibril interfacer 1 (EMILIN-1) is very weakly present in the ventricularis and spongiosa, as compared to a stronger expression in the heart muscle tissue.

### Valvular interstitial cells

**Figure 5:**
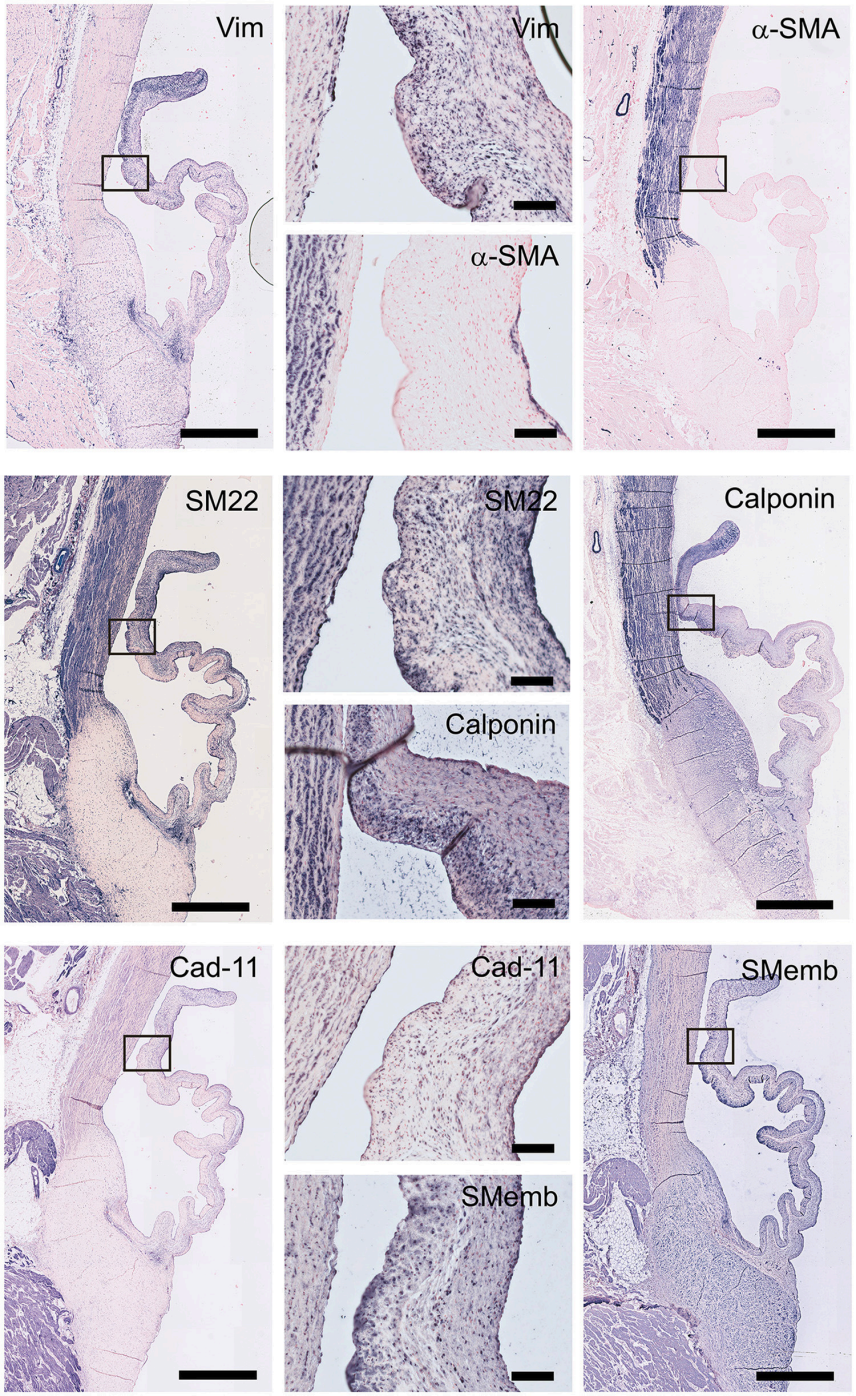
Valvular interstitial cell (VIC) markers. Tile scans of sheep aortic valve sections (left and right columns), with high magnification zooms (middle column) as indicated. Valves were stained with antibodies against vimentin, α-smooth muscle actin (α-SMA), SM22, calponin, cadherin-11 (Cad-11), and the embryonic form of myosin heavy chain (SMemb). Scale bars, 1 mm (tile scans) or 100 μm (zooms).

To characterize VIC phenotypes, several smooth muscle and mesenchymal markers are included in the antibody panel (Figure 5). The intermediate filament vimentin shows extensive staining in almost all cells in the leaflet, with particularly abundant presence of vimentin-positive cell in the tip of the leaflet. On the other hand, the valve leaflet contains only a very limited number of alpha-smooth muscle actin (α-SMA) positive cells below the endothelial layer on the ventricularis side of the leaflet. In contrast, the smooth muscle cells (SMCs) of the aortic wall and microvasculature are highly positive for α-SMA. Another marker for mature differentiated SMCs, SM22, strongly stains the aortic wall, heart muscle cells, microvasculature, and connective tissue. In the leaflet, SM22 displays a similar staining pattern as vimentin. The calponin antibody stains mainly the aortic SMCs, the microvasculature, and the cells in the fibrosa and tip of the leaflet. Cadherin-11 (as mesenchymal marker for VICs) is expressed in VICs throughout the leaflet, but especially in the ventricularis. Additionally, this antibody stains cells of the aorta and microvasculature and shows expression in the heart muscle. Non-muscle myosin heavy chain (SMemB), produced by activated mesenchymal cells, stains the VICs throughout the leaflet, the endothelial lining, as well as the cells of the aorta.

### Endothelial cells

**Figure 6:**
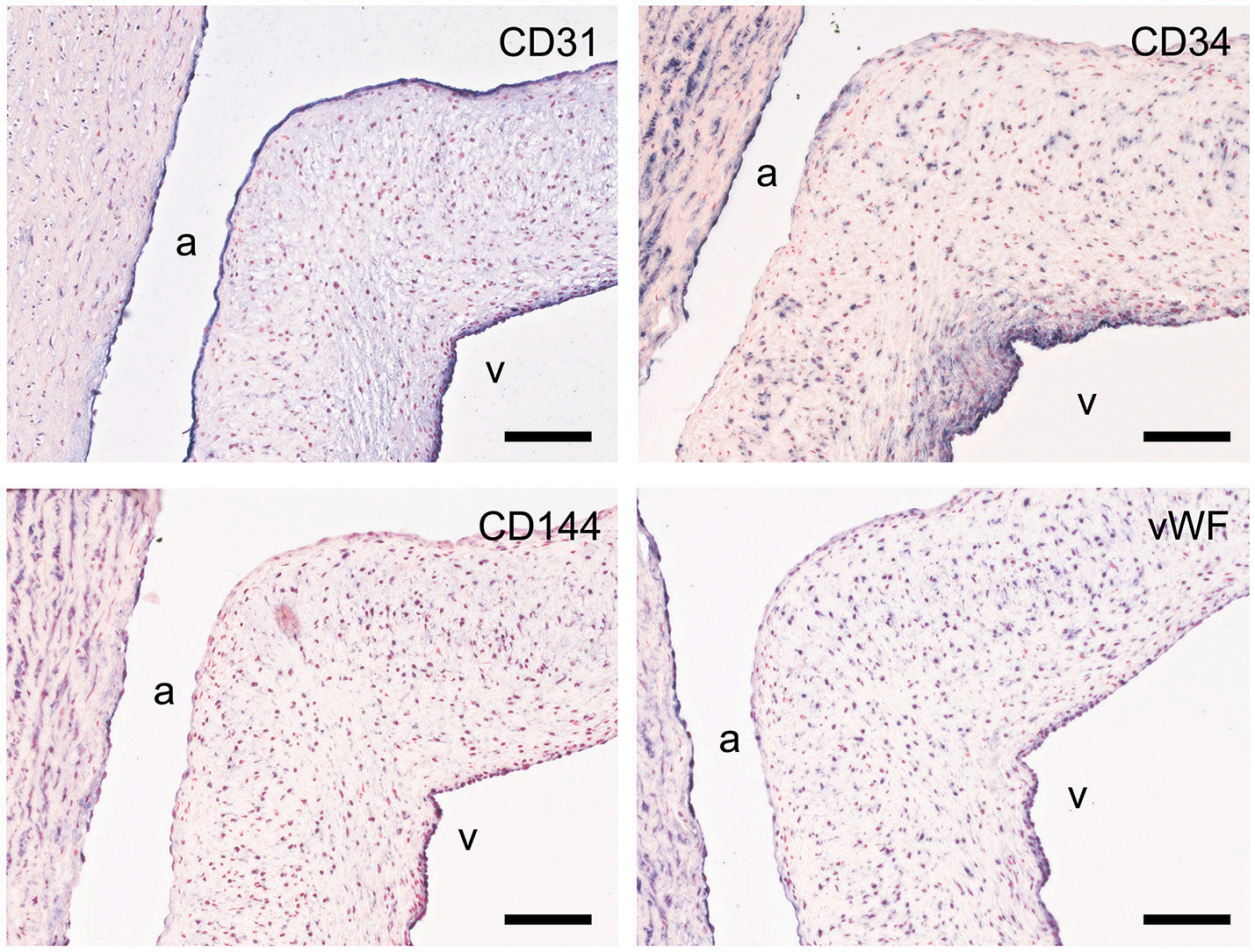
Valvular endothelial cell (VEC) markers. Sections of the sheep aortic valve leaflet stained with antibodies against the endothelial cell markers CD31, CD34, VE-Cadherin (CD144), or von Willebrand Factor (vWF). Represented are the aortic wall and a section of the valvular leaflet (near the free edge), with “a” and “v” indicating the aortic and ventricular sides of the leaflet, respectively. Scale bars, 100 μm.

CD31, CD34, von Willebrand factor (vWF), and vascular endothelial cadherin (VE-cadherin; CD144) were used to assess the endothelial cell function (Figure 6). All studied markers show expression in the aortic endothelial cells. CD31 specifically stains all endothelial cells of the valvular leaflet, while CD34, VE-cadherin, and vWF have differential expression in the endothelial cells on the ventricular side of the leaflet. CD34 is also present in VICs (more abundant in the ventricularis) and the aortic wall. VE-cadherin and vWF also show some modest staining of individual VICs and SMCs within the aortic wall.

### Proliferation and apoptosis

**Figure 7:**
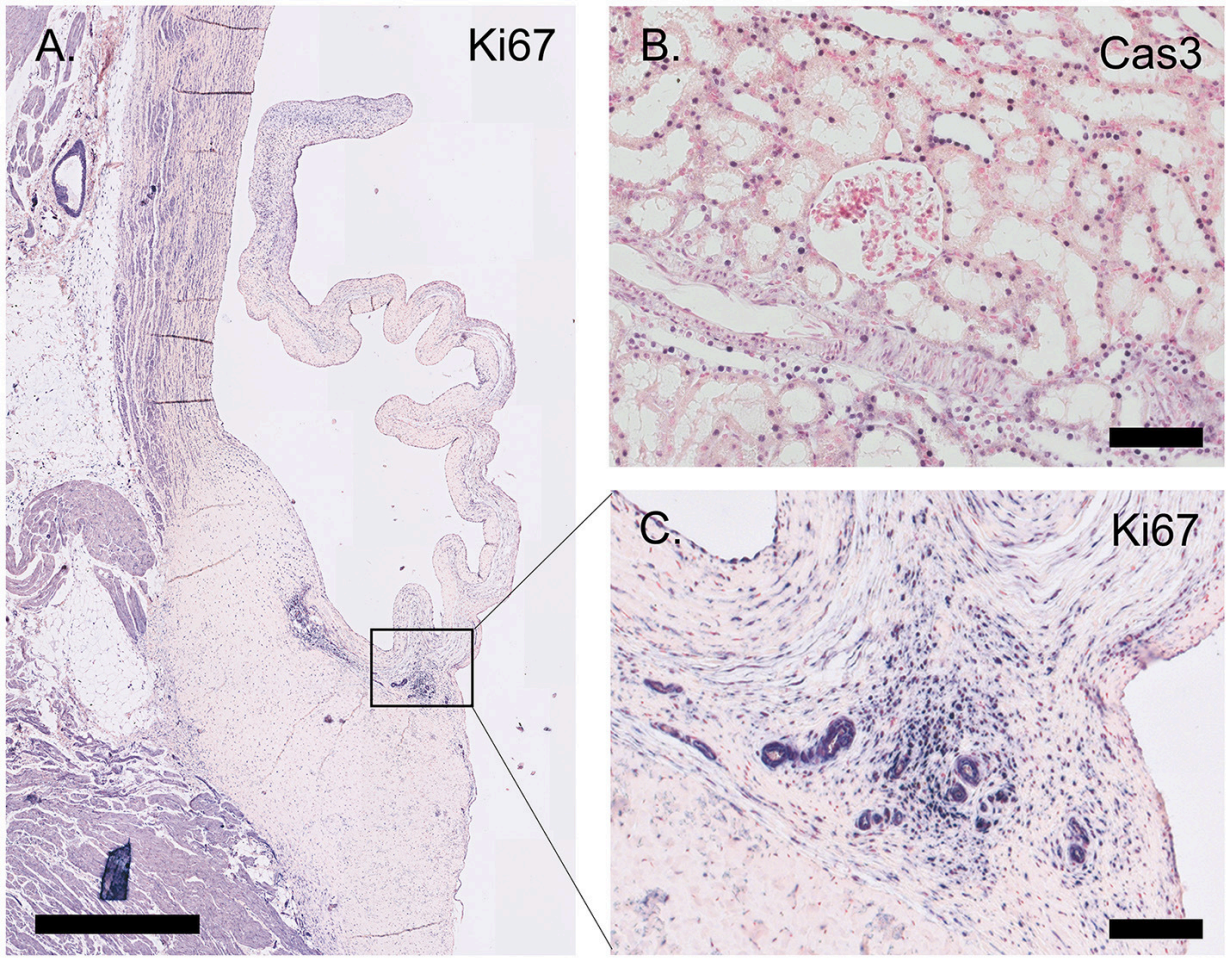
Proliferation and apoptosis markers. **(A)** Tile scan of an ovine aortic valve stained with the proliferation marker Ki67. **(B)** Section of the ovine kidney stained against the apoptosis marker cleaved caspase-3 (Cas3). **(C)** Zoom of the Ki67-labeled aortic valve demonstrating abundant Ki67 expression in the vascularized hinge region. Scale bars, 1 mm **(A)** and 100 μm **(B, C)**.

The proliferation marker Ki67 is abundantly expressed throughout the valve leaflet, predominantly staining VICs at the hinge and the tip of the leaflet (Figure 7). Apoptosis is evaluated using an antibody against cleaved caspase-3 (Cas3). Using this antibody, apoptosis was not observed in the valvular leaflet, but was detected in localized sections of the ovine renal tubuli (Figure 7).

### Calcification

**Figure 8:**
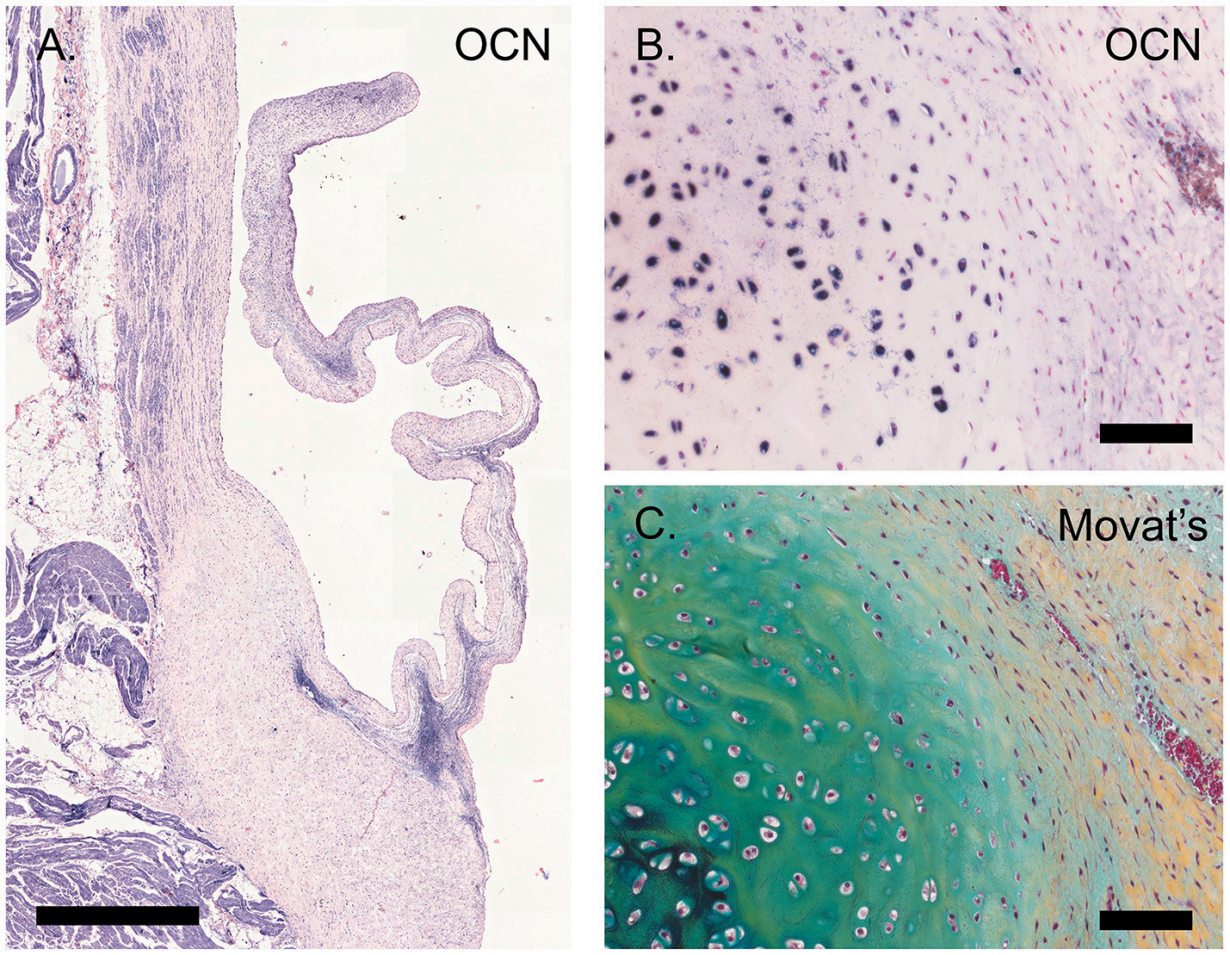
Calcification. **(A)** Tile scan of a healthy ovine aortic valve stained with osteocalcin (OCN). **(B)** Section of the ovine pulmonary wall in which the onset of calcification was detected, stained against OCN. **(C)** Russell Movat's pentachrome staining of the same section of pulmonary wall revealed abundant presence of proteoglycans (in blue-green), colocalizing with OCN expression. Scale bars, 1 mm **(A)** and 100 μm **(B, C)**.

Osteocalcin expression was detected in chondrocyte-like cells in a section of an ovine pulmonary leaflet in which the onset of calcification was observed (Figure 8). Although no calcium was detected in this region using alizarin red staining (data not shown), osteocalcin expression in this portion of the valve colocalized with strong proteoglycan presence, indicative of cartilage-like tissue, as evident from Russell-Movat pentachrome staining.

### Inflammatory cells

**Figure 9:**
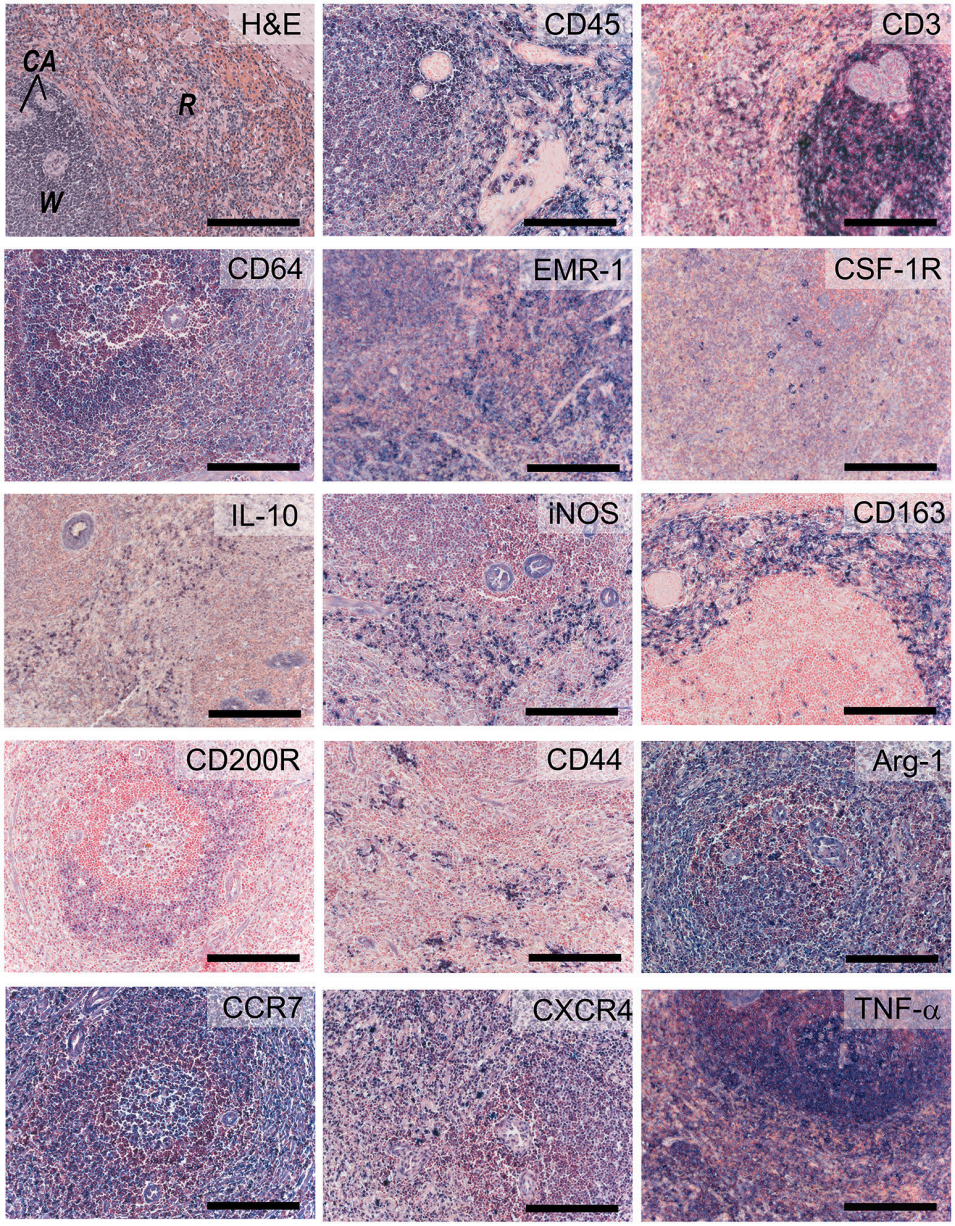
Inflammatory markers. Sections of activated ovine spleen, isolated from a sheep with endocarditis. Hematoxylin and eosin (H&E, top left) staining reveals the characteristic splenic red pulp (R) and white pulp (W), with the central arterioles (CA). Sections are stained with antibodies against CD45, CD3, CD64, EGF-like module-containing mucin-like hormone receptor-like 1 (EMR-1), colony stimulating factor-1 receptor (CSF-1R), interleukin-10 (IL-10), inducible nitric oxide synthase (iNOS), CD163, CD200R, CD44, Arginase-1 (Arg-1), CCR-7, CXCR4, and tumor necrosis factor-α (TNF-α). Scale bars, 200 μm.

The signs of inflammation are not observed in our non-diseased ovine valves. We therefore validated inflammatory markers using the spleen from a sheep with endocarditis (Figure 9). CD45 is abundantly expressed by all leukocyte cells, but not erythrocytes or the mesenchymal cells in the trabeculae and arterioles. CD3 staining is mainly observed in the germinal centers of the white pulp, with positive cells scattered in the mantle and marginal zones. CD64 and EGF-like module-containing mucin-like hormone receptor-like 1 (EMR-1) stain macrophages in both the red and white pulp, whereas colony stimulating factor-1 receptor (CSF-1R) displays a sparse staining of specific subpopulations of macrophages in both red and white pulp. IL-10, iNOS, and CD163 predominantly stain positive for the splenic tissue macrophages in the red pulp. Additionally, some isolated CD163- and iNOS-positive cells are noted in the white pulp. CD200R displays a specific staining in the marginal zone of the white pulp, whereas CD44 stains lymphocytes and myeloid cells predominantly in the red pulp. Excessive immune reactivity was observed for arginase-1 (Arg-1), CCR-7, and tumor necrosis factor-alpha (TNF-α).

## Discussion

Limited data is available on the *in vivo* processes that determine the regeneration and long-term functional integration of TE heart valves upon implantation. These require incorporation of inflammatory and developmental processes that drive neotissue formation toward functional regeneration or fibrotic repair. Since histochemical evaluation of explants is typically limited to basic histology, due to a lack of commercially available sheep-specific antibodies, the aim of this study was to establish, validate, and optimize a comprehensive immunohistochemical panel of antibodies for ovine tissues to study TE heart valves. This panel includes antibodies against structural ECM proteins (e.g., collagens, proteoglycans), ECM-related proteins (e.g., periostin, MMPs, TGF-β isoforms), and proteins responsible for elastogenesis, as well as cell phenotypic markers for VICs, ECs, inflammatory cells, and relevant cell function markers (e.g., proliferation, apoptosis, calcification).

The immunohistochemical analysis of explanted TE heart valves in preclinical studies is typically limited to a few antibody stainings assessing VIC activation and endothelial coverage (e.g., α-SMA and CD31). Nevertheless, several recent studies concerning *in situ* cardiovascular TE have employed antibodies to characterize more specific functions, for example the regenerative processes in decellularized allogeneic heart valves (15), the occurrence of elastogenesis in synthetic *in situ* TE heart valves (24), and the somatic growth of an *in situ* TE pulmonary artery in lambs (44). The need for a sheep-specific antibody panel for cardiovascular research has previously been proposed by De Visscher et al. who reported on a panel of immunohistochemical and immunofluorescent stains for sheep (45). Although highly relevant, the selected antibodies were tested on formalin-fixed frozen sections of various ovine tissues, such as artery and skin, but not on native ovine heart valves. Building on these previous reports, to the best of our knowledge, the described panel is the first comprehensive panel of 47 antibodies, validated against native ovine heart valves, which enables a complete and detailed evaluation of TE heart valve explants from sheep studies.

In the selection of antibodies for our panel, we took inspiration from various studies describing the morphology, ECM composition and cellularity of native human heart valves throughout development and adult stages (39–42, 46). As anticipated, we observed several clear differences between human and ovine valves. Most strikingly, the ovine leaflets, both pulmonary and aortic, displayed a much more defined transition between the valvular layers, higher cellularity and less elastin expression when compared to the human valves. Human valves instead often demonstrate incorporation of collagen fibers and elastin network within the other layers (47). These differences observed between human and ovine valves could partly be caused by disease (48) and a discrepancy in age, since recent studies have shown that human valves continuously develop (40, 49) and degenerate throughout life. The ovine aortic valves used in this study were derived from 1 year old sheep, which translates to human adolescence (15–19 years of age), while the human valves were derived from a 38-year old human donor. The human valves were cryopreserved before further processing, which may have introduced freezing artifacts. Moreover, the sections of the human valves were thicker compared to the sections of the ovine valves (10 μm and 5 μm, respectively), which could lead to variations in intensity of the pentachrome staining and antibody expression. Previous research demonstrated significant differences between human- and sheep-derived VIC-like cells, in terms of ECM synthesis and cellular proliferation (50). Similarly, here we observed a notably high amount of proliferating VICs in the ovine aortic leaflet, as determined by Ki67 staining. In contrast, proliferation and overall cell turnover were very low in human adults as compared to fetal valves (39). Notably, the observed protein expression was remarkably similar between the various ovine leaflets (both aortic and pulmonary) that were included in the validation.

All tested structural proteins exhibit specific staining patterns, as summarized in Table 2. Collagen is an essential load-bearing component of valvular ECM that contributes significantly to the mechanical strength of the tissue. As previously reported, collagen type I is mainly observed in the fibrosa, while collagen type III expression is more diffused throughout the leaflet (40, 42). Periostin co-localizes with collagen type I as described before (51), supporting its role in collagen deposition and maturation (52–54). Glycoaminoglycans are found throughout the heart valves (55). As both decorin (56) and biglycan (57) are involved in collagen fiber formation, and collagen type III has a critical role in fibrillogenesis (58), it is not surprising that the expression patterns of these proteins are mostly overlapping. Versican is mainly observed in the ventricularis, which supports the important role of this protein in the assembly and organization of elastin (59). Complementary to the matrix proteins, our panel includes collagenases (MMP1, MMP13) and gelatinases (MMP2, MMP9) as important proteolytic enzymes involved in physiological matrix turnover and remodeling. Human heart valves display specific expression patterns of MMPs and tissue inhibitors of metalloproteinases (TIMPs), which differ between the four different heart valve (60). In this respect, it would be relevant to expand the current panel with antibodies against various TIMPs, which have not been validated in our study.

**Table 2:**
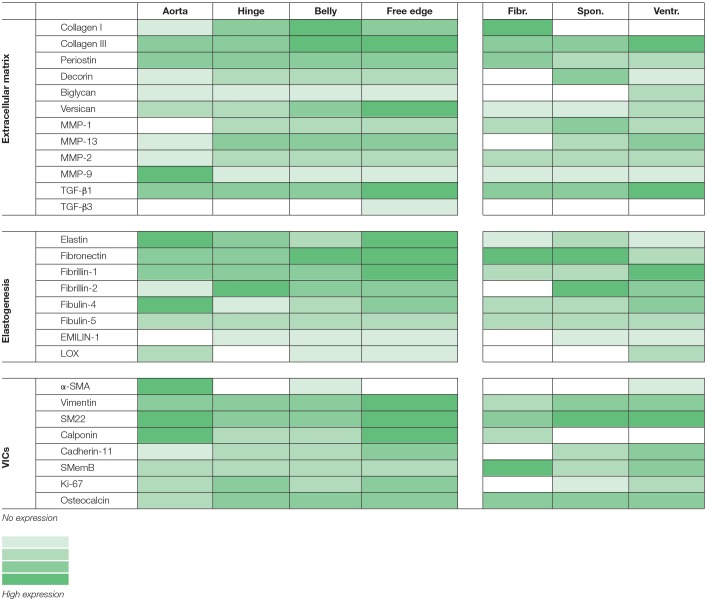
Summary of protein expression in the ovine aortic valve; categorized per region (aorta, leaflet hinge, belly, and free edge), or per leaflet layer (fibrosa, spongiosa, ventricularis).

One of the hallmarks of functional regeneration of heart valves is the formation of an elastic network, or elastogenesis. Elastin is mainly found in the ventricularis layer of healthy adult valves. It has been demonstrated that Verhoeff's method does not stain immature fibers, and thus, it is not efficient for evaluating the process of elastogenesis during valve development and remodeling. As delineated by Wagenseil and Mecham (61), the processes underlying elastic fiber formation are highly complex and require the interplay of various molecules. These include the core protein (tropo)elastin, microfibrillar proteins (fibrillin-1 and fibrillin-2) and various linking proteins, including EMILIN-1 and LOX. Other proteins involved include fibulin-4, which links the enzyme LOX to tropoelastin (62) and fibulin-5, which acts as a bridge between microfibrils and tropoelastin (63, 64). In addition, a fibronectin network is needed for the assembly of fibrillins, and consequently of microfibrils, that provides a microenvironment that controls tropoelastin/elastin arrangement and cross-linking processes (65). In addition to their essential role in elastogenesis, many of these proteins have been reported to have important signaling roles in pathophysiological tissue remodeling processes, relevant to TE (66, 67). In the present study, validation of the antibodies revealed specific expression patterns of elastogenesis-related proteins in the ovine heart valve leaflet (see Table 2). Fibrillin-1, for example, is not exclusively located in the ventricularis, but also spreads into the spongiosa, which is in accordance with previous findings by Votteler et al. for mature human heart valves (41). Similarly, although EMILIN-1 only shows weak staining, it is detectable in both the ventricularis and the spongiosa, which was also reported for human valves. One notable difference however, is the abundant expression of fibrillin-2 in the ovine leaflet, and particularly in the leaflet tip, compared to undetectable levels of fibrillin-2 in adolescent and adult human valves, as reported in the study by Votteler et al. This points to species-differences in fibrillin-2 expression, which may be attributed to the aforementioned differences in ECM synthesis and activity of ovine VICs when compared to human VICs.

Healthy adult heart valve leaflets contain mainly a quiescent population of VICs of fibroblast-like phenotypes that maintain valve homeostasis and structural leaflet integrity (39, 48, 68). In addition, SMCs and activated myofibroblasts are interspersed between the ECM layers (39, 69). α-SMA expressing myofibroblasts are present throughout the entire leaflet mostly during disease and development (39, 48). Myofibroblasts produce and secrete most of the surrounding ECM, particularly collagen, whereas a small subpopulation of mature SMCs (marked by SM22, Calponin) and dedifferentiated SMCs (marked by SMemb) secrete MMPs and TIMPs. Fibroblast-like VICs express general mesenchymal markers, such as vimentin and CD44, the latter being required to remodel ECM (70). Quiescent VICs can be activated by environmental cues and stimulated by valvular endothelial cells (VECs) (71), leading to differentiation into myofibroblast-like α-SMA-positive VICs. These activated VICs are important for valve remodeling. However, continuous and uncontrolled VIC activation in adult valves is associated with valve diseases resulting in fibrosis and calcification. For heart valve TE, the VIC phenotypic state can reflect the current remodeling demands of the valvular tissue at a particular stage of the process of heart valve formation. In a comparative study of native and TE heart valves, TE valves displayed persistent expression of VIC activation markers when compared to quiescent VICs in native valves (69). Hence, to account for the complex and dynamic phenotypic manifestations of VICs, a range of SMC- and fibroblast-associated markers has been included in our panel of antibodies.

The ovine valve analyzed in our study displayed a notably high expression of SMC markers (see Table 2). Given that the aortic valve we used for antibody validation was derived from a sheep that had undergone pulmonary valve replacement with a synthetic valve, the observed expression of SMC markers may be indicative of VIC activation as a side-effect of that intervention. Moreover, pronounced osteocalcin expression was detected in the leaflet, which may be indicative of the presence of osteoblast-like VICs as a result of persistent VIC activation and dysregulated apoptosis, as previously reported (72). Correspondingly, in human valves, expression of SMC markers and co-activators has been correlated to valve calcification (73), suggesting that markers such SM22 and calponin could be expressed by other cell types within heterogeneous VIC populations. In addition, recent studies demonstrated that cadherin-11 is not only involved in embryonic heart development and valve maturation (74, 75), but also associated with calcification of aortic valves (76). Another useful marker for cardiovascular (micro)calcifications, not included in our antibody panel, could be sortilin, a sorting protein and a key regulator of SMC calcification via its recruitment to extracellular vesicles (77).

With respect to VECs, of the markers we tested, CD31 demonstrates the most specific endothelial staining on the ovine leaflet. Apart from VECs, CD34 is a well-known marker for hematopoietic progenitor cells, and when combined with other markers such as CD45 and collagen type I, can be used to identify fibrocytes (78). Of note, CD34, vWF and VE-Cadherin were mostly expressed in VECs on the ventricular side of the leaflet, the side exposed to high flow, but not the aortic side of the leaflet. Importantly, although not validated here, the selected VIC and VEC antibodies (e.g., α-SMA and CD31) do allow for double-label immunofluorescent staining to assess endothelial-to-mesenchymal transition (EndMT). This is highly relevant for evaluating heart valve TE, as basal levels of EndMT may contribute to the replenishment of VICs as part of physiologic valve remodeling throughout postnatal life (79, 80), and VEC-VIC interactions are important for maintaining valve homeostasis and the prevention of osteogenesis (72).

In addition to the valve-associated markers that constitute our panel of antibodies, we selected antibodies to characterize immune cells and the inflammatory state of a tissue, such as the pan-leukocyte marker CD45, lymphocyte marker CD3, and pro- and anti-inflammatory cytokines (e.g., TNF-α and IL-10, respectively). Inflammation is well-established to be a critical regulator of pathophysiological valvular remodeling (81). For TE approaches, the host immune response and the inflammation induced by the implanted heart valve graft is one of the most critical determinants of successful and functional valve integration. In fact, recent *in situ* cardiovascular TE approaches rely on the notion that the host inflammatory response can be used as the driver of endogenous tissue regeneration when harnessed properly [reviewed in (13)].

The primary target cells we focused on in the current study were macrophages. As in normal wound healing, macrophages play an important role in regeneration, via the cross-talk with (myo)fibroblasts and other immune cells, such as T cells. Depending on their polarization state, macrophages mediate the formation and remodeling of new tissue by secreting essential growth factors and cytokines that either inhibit or promote functional tissue formation. Pro-inflammatory (M1) macrophages (characterized by iNOS, CCR-7) maintain inflammation state via the secretion of e.g., TNF-α, while alternatively activated (M2) macrophages (characterized by CD163, Arg-1, CD200R) secrete high levels of IL-10 and TGF-β to suppress the inflammation, and contribute to tissue repair, remodeling, vasculogenesis and retain homeostasis (82). Previous studies suggest that the M2/M1 macrophage ratio in early phases after implantation can be used as a predictor for long-term tissue outcome in natural biomaterials (83). Albeit practical, the M1-M2 paradigm is highly simplified. More realistically, macrophages form a very heterogeneous cell population with a broad range of (overlapping) M1–M2 characteristics (84). As a consequence, macrophage characterization is not straightforward and marker expression of macrophages specific for sheep has not yet been described. Of interest, Griebel et al previously reported cross-reactivity of a range of CD antigens for sheep lymphoid and myeloid cells (85). However, that study was focused on flow cytometry and it has to be tested whether those antibodies are suitable for use as immunohistological staining on paraffin or frozen sections. In our study, the spleen samples were obtained from a sheep with endocarditis, and thus represented an activated inflammatory state. The spleen tissue contained heterogeneous pools of macrophages, where M2-like macrophages were positive for CD163 and CD200R, whereas M1 macrophages expressed iNOS. Arginase and CCR7, reported as M2 and M1 markers, respectively, in different species, were abundantly expressed in spleen samples. None of the markers we tested represented a suitable pan-macrophage marker for sheep, for example analog to human CD68. Multiple anti-CD68 clones were tested on inflamed lung and spleen tissues under different antigen retrieval conditions, leading to unsatisfactory results (data not shown). EMR-1, the human homolog to F4/80 in mouse, could serve as a general marker. Although in humans expression of EMR-1 is restricted to eosinophils (86), we observed a less specific expression pattern in the ovine spleen. CSF-1R was tested as an alternative macrophage marker, being previously described as a marker committed to the mononuclear phagocytic lineage (87). In our panel, CSF-1R displays a highly specific expression pattern restricted to a small number of individual cells in the spleen. CD64, a classical monocyte- and macrophage-associated marker, was identified to be the most reliable pan-macrophage marker in sheep. To further discriminate between monocytes and macrophages, various CD14 antibodies were tested, but no positive immune reaction was observed in the ovine tissues (data not shown). Of note, CD44 is known to be highly induced in macrophages at the onset of fusion and, as such, can serve as an excellent marker for multinucleated giant cells (88). Taken together, there are very few unique macrophage markers and a combination of markers is required to identify the specific macrophage phenotype. For TE constructs, combinations of different anti-macrophage antibodies could give a spatiotemporal indication of the status of inflammation and wound healing. Moreover, macrophage polarization state is governed via the cross-talk with other immune cells, such as T helper cells, which have been reported to play an essential role in tissue regeneration as well (89). Consequently, markers to distinguish T helper 1 and T helper 2 cells (e.g., CXCR3 and CCR8, respectively) would be valuable additions to the antibody panel as described in our study.

From a translational point of view, our results indicate substantial differences between human and ovine valves that should be taken into account in the preclinical testing of valve replacements (e.g., tissue-engineered heart valves) using the sheep model. Particularly, whereas the expression of activated VIC markers (e.g., SM22, calponin, periostin, osteocalcin) are typically associated with pathological events in human valves, the abundant expression of these markers in the native ovine valves as observed in this study, suggests that these markers are not necessarily representative of adverse remodeling in TE heart valves when evaluated in sheep. On the other hand, the observed continuous expression of these same markers may be one of the factors that underlie the increased tendency for calcification of sheep. All in all, while the sheep still represents the animal model of choice for the preclinical evaluation of heart valve substitutes, our findings imply that data obtained from preclinical studies in sheep, particularly in terms of tissue organization and remodeling and VIC activation, should be interpreted in the correct context and with the appropriate caution, as data may not translate directly to the human situation.

## Conclusion

The field of heart valve TE has made great progress over the last decades. However, little mechanistic data is available on the *in vivo* inflammatory and remodeling processes underlying functional tissue regeneration of TE heart valves. The comprehensive sheep-specific panel of antibodies described in this study could serve for the detailed evaluation of the regenerative processes including ECM formation/remodeling, elastogenesis, VIC, and VEC phenotyping, and inflammation, when assessing TE heart valves implanted in sheep, the designated preclinical model.

## Author contributions

SD, DvG, EA, AvdB, and AS contributed to the investigation, methodology, and validation of the study. SD, DvG, and AS contributed to visualization of the data. AD-M and AS conceptualized and supervised the study, and AS acquired the funding. SD, DvG, and AS prepared the original draft. EA critically read and edited the final version of the manuscript. All authors reviewed and edited the manuscript and agreed to be accountable for all aspects of the work involved in preparing this manuscript.

### Conflict of interest statement

The authors declare that the research was conducted in the absence of any commercial or financial relationships that could be construed as a potential conflict of interest.
